# High Dose of Caffeine Mouth Rinse Increases Resistance Training Performance in Men

**DOI:** 10.3390/nu13113800

**Published:** 2021-10-26

**Authors:** Raci Karayigit, Mitat Koz, Angela Sánchez-Gómez, Alireza Naderi, Ulas Can Yildirim, Raúl Domínguez, Fatih Gur

**Affiliations:** 1 Faculty of Sport Sciences, Ankara University, Gölbaşı, Ankara 06830, Turkey; mitat.koz@ankara.edu.tr (M.K.); ulas.can.yldrm.ucy@gmail.com (U.C.Y.); 2 Department of Nursing Pharmacology and Physiotherapy, Faculty of Medicine and Nursing, University of Córdoba, 14000 Córdoba, Spain; asgomez@uco.es; 3 Department of Sport Physiology, Boroujerd Branch, Islamic Azad University, Boroujerd 6915136111, Iran; Naderi_a@yahoo.com; 4 Departamento de Motricidad Humana y Rendimiento Deportivo, Universidad de Sevilla, 41013 Sevilla, Spain; 5 Studies Research Group in Neuromuscular Responses (GEPREN), University of Lavras, Lavras 37200-000, Brazil; 6 Faculty of Sport Science, Pamukkale University, Pamukkale, Denizli 20000, Turkey; fatihgur@pau.edu.tr

**Keywords:** ergogenic aids, mouthwash, supplement, strength, muscular endurance

## Abstract

Caffeine mouth rinsing (CMR) has been shown to enhance exercise performance. However, no studies have analyzed the effects of different dosages of CMR on muscular performance. Therefore, the purpose of this study was to examine the effects of different dosages of CMR on strength (bench press 1 repetition maximum (1-RM)) and muscular endurance (60% of 1-RM repetitions to failure) in resistance-trained males. Fourteen resistance-trained males (age: 23 ± 2 years, height: 179 ± 3 cm, body mass: 83 ± 4 kg, BMI: 17 ± 2 kg/m^2^) completed four conditions in random order. The four conditions consisted of a mouth rinse with 25 mL solutions containing either 1% (250 mg) of CMR (low dose of CMR: LCMR), 2% (500 mg) of CMR (moderate dose of CMR: MCMR), 3% (750 mg) of CMR (high dose of CMR: HCMR) and sweetened water (placebo: PLA) for 5 s prior to a bench press strength and muscular endurance test. Maximal strength, muscular endurance, heart rate (HR) and ratings of perceived exertion (RPE) were recorded for each condition. There were no significant differences in strength (*p* = 0.30) and HR (*p* = 0.83) between conditions. HCMR significantly increased muscular endurance performance (*p* = 0.01) and decreased RPE values (*p* = 0.01). In conclusion, CMR did not affect bench press 1-RM strength performance, but muscular endurance responses to CMR seems to be dose-dependent.

## 1. Introduction

Caffeine is one of the most used nutritional ergogenic aid by athletes [1] and considered effective in improving cardiorespiratory endurance [2], movement velocity [3], power [4] and muscular endurance during resistance exercises [5,6]. The exact mechanisms, likely to be multifactorial, by which it shows benefits in neurotransmission, perceived exertion and arousal, are mediated by adenosine receptor (A_1_, A_2A_, A_2B_) antagonism [7]. Furthermore, evidence still seems conflicting, with some research demonstrating no benefits in muscular strength [8] and endurance [9] performance after caffeine ingestion. In research studies, caffeine is generally consumed in moderate (3 mg/kg) to high (6 mg/kg) doses, with 9 mg/kg being uncommon [10]. Grgic et al. [11] also concluded that caffeine doses of 4 and 6 mg/kg enhanced barbell bench press strength with a meaningful additive trend for the erogenicity of different doses of caffeine. However, intake of caffeine might lead to some side effects during or after exercise, such as anxiety, headache, gastrointestinal discomfort and insomnia [12]. Therefore, alternative forms of caffeine usage that may mitigate its common side effects are relevant.

Caffeine mouth rinsing (CMR) has been proposed as an effective alternative to enhancing exercise performance without promoting side effects [13,14]. CMR strategy involves 5–20 s of rinsing in the mouth without further ingestion. Two potential mechanisms of CMR are alleged to be involved in ergogenic effects. First, caffeine binds with adenosine receptors found in the mouth and increases neurotransmitters’ release and motor unite firing rates [15]. Secondly, bitter taste receptors located in the oral cavity directly connected to the brain regions responsible for information processing and reward [16] are activated when expose to caffeine, enhancing mental alertness through dopamine transmission [17]. CMR has been tested in different sports modalities, such as sprint cycling [18], 30 min cycling endurance [19] and running-based aerobic endurance tests [20]. Melo et al. [21] stated that 10 s of 300 mg of CMR was effective to improve endurance performance during exercise lasting more than 60 min. Conversely, 10 s of 35 mg of CMR was reported to have no impact on endurance cycling time-trial performance [22]. Heterogeneity in the test protocols (concentration, mouth rinse time, habitual consumption) were concluded by a recent review to influence the efficacy of CMR on exercise performance [23].

The main cause of fatigue during maximal resistance exercise is related to reduced neural drive [24]. Moreover, Smilios et al. [25] has suggested to muscle electrical activity may increase up to a point to overcome fatigue during moderate load muscular endurance session. In this regard, CMR can be used to enhance central drive and has the potential to increase strength or muscular endurance performance. However, only one study [26] to date has assessed the effect of CMR on muscular performance and reported no benefits on one repetition maximum (1-RM) and 60% of 1-RM repetitions to failure performance. Despite using the same concentration of caffeine as previous rinsing studies [18,21], the authors suggested for future research that there may be a benefit in increasing to a 300 mg dose and including an additional rinse between maximal strength and muscular endurance assessments [26]. In line with the occupancy hypothesis [27], the greater the dosages of caffeine, the more adenosine and taste receptors within the mouth can be stimulated, and thus help to improve muscular performance. However, no research, up to now, has examined whether a possible dose–response relationship exists between the concentration of the CMR solution and exercise performance. Therefore, the aim of the present study was to examine the effects of various doses of CMR on 1-RM strength and 60% of 1-RM muscular endurance performance. We hypothesized that benefits of CMR on strength and muscular endurance would increase in a dose-dependent manner.

## 2. Materials and Methods

### 2.1. Participants

Fourteen healthy, non-smoker resistance-trained males participated in this study voluntarily (age: 23 ± 2 years, height: 179 ± 3 cm, body mass: 83 ± 4 kg, BMI: 17 ± 2 kg/m^2^, resistance training experience: 3 ± 1 years). All participants declared that they were not using any nutritional supplements that could affect muscular biology and performance in the 3 months previous to the start of the study. Resistance-trained males had at least 4 years of resistance-type exercise participation, three times per week for the previous year and included upper body exercises such as bench press in their training schedule. Daily caffeine intake level was measured via an adapted version of the daily caffeine intake level questionnaire suggested by Buhler et al. [28], under the supervision of a qualified nutritionist. Household measures were used to assess the quantity of caffeine-containing food during per day, week and month. All participants were very low caffeine users (12 mg/day) according to Filip et al.’s [29] classification. Inclusion criteria were as follows: (a) non-smoker, (b) very low caffeine consumer, (c) free from neuromuscular and musculoskeletal disorders and (d) able to perform successful bench press exercises with load equal to 100% of their current body mass. Participants were made fully aware of the procedures, including any risks of participation in the study, before providing written informed consent. The experimental methods were undertaken in accordance with the Declaration of Helsinki and approved by Sinop University, Human Research Ethic Committee (2021/114).

### 2.2. Study Design

**Figure 1:**
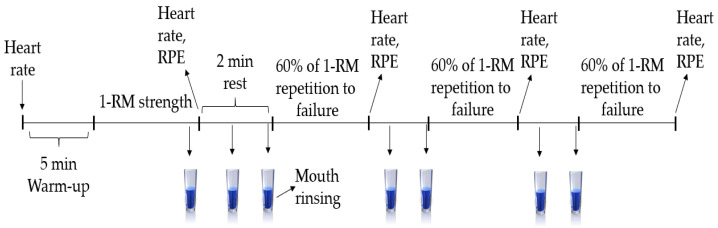
Schematic diagram of experimental protocol.

Following one session of familiarization, participants were randomized in a cross-over, counterbalanced, double-blind design to four experimental conditions: mouth rinsing with a 1% caffeine solution (LCMR), 2% caffeine solution (MCMR) or 3% caffeine solution (HCMR), and water as a placebo (PLA). The 1% (250 mg) and 2% (500 mg), but not 3% (750 mg), caffeine solutions were used in previous studies [30,31] and represent low (3 mg/kg), moderate (6 mg/kg) and high (9 mg/kg) doses, respectively, for athletes with average body mass [10]. Testing sessions were performed on different days (48 h apart) at the same time slot to avoid any effect of circadian rhythm. Upon arrival at the laboratory (07:00–08:00) following a 10 h night fasting, participants performed warming up exercises for 5 min on a treadmill followed by determination of bench press maximal strength and 3 sets of muscular endurance, interspersed by a standardized 2 min passive rest. Ratings of perceived exertion (RPE) (6–20 Borg) [32] and heart rate (HR) (Polar Team 2 telemetric system, Kemple, Finland) were determined at various time points throughout the test protocol (Figure 1). CMR and placebo solutions were prepared by a researcher who did not participate in data collection to ensure double blinding. Participants were also requested to abstain from caffeine-containing foods and drinks, as well as alcohol ingestion and strenuous physical activity in the 24 h leading up to each visit. On the day preceding the first test session, participants recorded their 24-hour dietary intake and then these recordings were photocopied by a researcher and handed back to the participants again to replicate this diet before subsequent sessions. Furthermore, adherence to diet and avoidance procedures (caffeine, alcohol and vigorous physical activity 24 h before each session) were checked verbally before each session.

### 2.3. Maximal Strength (1-RM) and 60% of 1-RM Muscular Endurance Test Protocol

Before commencing 1-RM determination on bench press, participants performed ten repetitions with a 20 kg weight to warm up, followed by resting for 1 min and then 3–5 repetitions adding 10% more weight, followed by 2–3 repetitions approaching near maximum effort with 2 min of passive rest. Three minutes of passive rest were provided to participants prior to the first 1-RM attempt. If successful, the weight was increased by 10% with a 3 min passive rest; otherwise, the weight was decreased by 2.5–5%. Bench press 1-RM strength was measured in 3–5 attempts as previously described [33]. After 1-RM determination, 2 min passive rest was given to adjust the weight for 60% of 1-RM individually, which was used for the 3 sets of muscular endurance tests separated by 2 min of passive rest. A certified personal trainer checked the test protocol and provided feedback if necessary to standardize the bench press exercise technique. The number of repetitions during 60% of 1-RM bench press endurance for each set was recorded and used as a muscular endurance index. Repetition speed (2 s for eccentric and concentric phases) during both 1-RM strength and muscular endurance tests was stabilized via a metronome. Three criteria were established to terminate the muscular endurance test protocol: (1) participants could not accomplish an extra repetition with appropriate technique and posture; (2) unable to perform repetitions at the same time with the metronome for three consecutive repetitions; (3) voluntarily terminated the repetition.

### 2.4. Mouth Rinsing Protocol

During each test session, participants were given a 25 mL bolus of either 1% (250 mg) as a low dose of caffeine mouth rinse (LCMR), 2% (500 mg) as a moderate dose (MCMR), 3% (750 mg) as a high dose (HCMR) or water (PLA). Solutions were rinsed around the buccal cavity for 5 s [18] and then expectorated into a plastic cup. Each solution was administered immediately before each attempt in the 1-RM strength test and at each min (2 times in total) between sets in the muscular endurance test. All solutions were flavored with 300 mg of sucralose and were similar in appearance. The same investigator prepared the solutions using electronic laboratory scales and distilled water at room temperature.

### 2.5. Statistical Analysis

All analysis was performed using the IBM SPSS statistics (version 22.0; IBM Corp., Armonk, NY, USA). Data are presented as mean ± SD. After a Shapiro–Wilk test approved distribution normally, two-way repeated measures analysis of variance (ANOVA) was used to examine the main effects for (1) condition, (2) time or set and (3) condition × time or set interaction. Mauchly’s test was performed to analyze sphericity followed by the Greenhouse–Geisser adjustment where required. Post hoc Bonferroni adjustment was performed if any main effects or interactions were identified. Intraclass correlation coefficients (ICC) were provided to determine the consistency of the four trials with conditions (two-way mixed model in consistency type). The effect sizes were assessed using partial eta squared (η^2^) as trivial (<0.10), moderate (0.25–0.39) or large (≥0.40). Statistical significance was set at *p* < 0.05.

## 3. Results

### 3.1. Strength (1-RM) and 60% of 1-RM Muscular Endurance Performance

**Figure 2:**
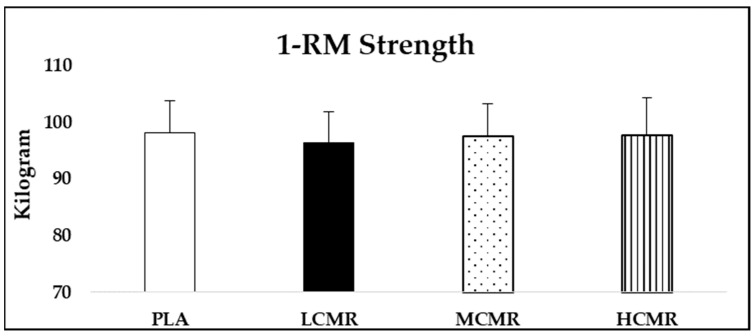
Mean (SD) bench press strength (1-RM) performance. PLA: placebo; LCMR: low dose of caffeine mouth rinse; MCMR: moderate dose of caffeine mouth rinse; HCMR: high dose of caffeine mouth rinse.

**Figure 3:**
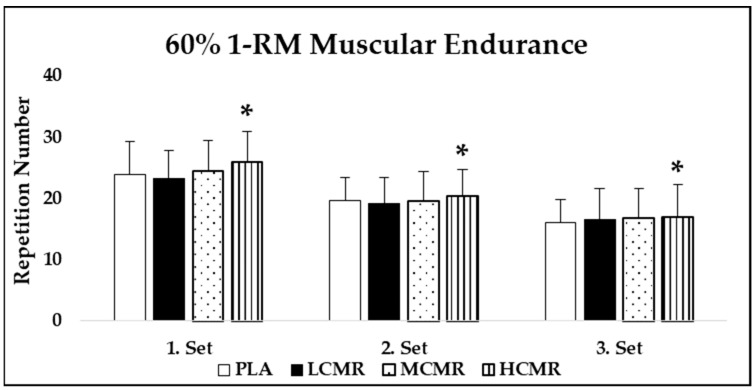
Mean (SD) 60% of 1-RM endurance performance. PLA: placebo; LCMR: low dose of caffeine mouth rinse; MCMR: moderate dose of caffeine mouth rinse; HCMR: high dose of caffeine mouth rinse. *: Significantly different from PLA and LCMR.

There was no main effect for condition (*p* = 0.30, η^2^ = 0.08) in 1-RM strength performance, meaning CMR did not affect this parameter (Figure 2). Furthermore, no condition × set interaction (*p* = 0.63, η^2^ = 0.05) was detected in 60% of 1-RM endurance performance, but there was main effect for condition (*p* = 0.01, η^2^ = 0.29). Post hoc analysis revealed that HCMR has significantly higher repetition numbers compared to PLA (*p* = 0.03) and LCMR (*p* = 0.01). There was no difference between HCMR and MCMR (*p* = 0.38). MCMR also was not different compared to PLA (*p* = 0.99) and LCMR (*p* = 0.46). Moreover, there was a main effect for time (*p* = 0.01, η^2^ = 0.79), as repetition numbers decreased from first to third sets (Figure 3). ICC, for 1-RM strength measurement, was 0.95, and for 60% of 1-RM endurance, ICC was 0.97, 0.92 and 0.90 for the first, second and third sets, respectively.

### 3.2. Heart Rate and Ratings of Perceived Exertion

HR did not differ between conditions (*p* = 0.83, η^2^ = 0.02) and no condition × time interaction was detected (*p* = 0.26, η^2^ = 0.09). However, as expected, HR values significantly increased with time (*p* = 0.01, η^2^ = 0.97) through the end of the test protocol. There was no condition × time interaction (*p* = 0.12, η^2^ = 0.12) for RPE values. However, the main effect for condition was significant (*p* = 0.01, η^2^ = 0.33). Bonferroni adjustment showed that HCMR significantly lowered RPE values compared to PLA (*p* = 0.04), LCMR (*p* = 0.01) and MCMR (*p* = 0.01). Furthermore, RPE values in all conditions increased with time (*p* = 0.01, η^2^ = 0.87) (Table 1).

## 4. Discussion

The main finding of the current research was that 5 s rinsing with a high (3%) dose of CMR has a significant benefit for bench press endurance performance. Furthermore, the composition of the solution has no effect on strength (1-RM) performance and HR values. However, a high dose (3%) of CMR significantly decreased RPE levels during 60% of 1-RM repetitions to failure performance.

Presence of caffeine, irrespective of dosages, in the oral cavity did not affect the 1-RM strength performance in the current study. If caffeine is ingested, it has been generally accepted that caffeine increases 1-RM strength performance in men [34] and women [35]. Furthermore, in a review of recent research [7], it was examined if there is a difference in caffeine’s effect on 1-RM strength between the upper and lower body and it was concluded that caffeine is ergogenic in both lower and upper body strength. This assertion was essentially confirmed by other studies [11,36] that reported an ergogenic effect of caffeine on 1-RM strength in the squat and bench press. On the other hand, sub-group analysis of a meta-analysis by Warren et al. [37] revealed that caffeine appears to improve strength primarily in the knee extensors and not in other muscle groups such as the forearm. The activation level during maximal voluntary contraction is normally lower for the knee extensors compared with upper body muscle groups (i.e., 85–95% vs. 90–99%) [38]. If caffeine improves strength performance by increasing motor unite firing rates, then there is a logical explanation of why upper body 1-RM strength in the current study did not respond to CMR because their activation level is already near 100% and there is no room for enhancement. Supportively, 10 s rinsing with a 300 mg of caffeine solution has no meaningful effect on bench press 1-RM strength performance [26]. In this regard, future studies should investigate the effect of CMR on lower and upper body strength performance in the same research design. Another potential explanation for the current and Clarke et al.’s [26] study demonstrating no improvement in the bench press 1-RM strength could be that mode of strength test may not be susceptible adequately to find any effect on performance. It can be speculated that benefits from CMR on 1-RM strength may disappear in day-to-day strength performance fluctuations (5%), which was shown previously [39]. Although high consistency (ICC = 0.95) appeared in the bench press 1-RM values in the current study, if a more sensitive method is used, such as isokinetic dynamometer, small benefits of a high dose of CMR can likely be detected.

High dose (3%) of CMR significantly enhanced three sets of 60% of 1-RM repetitions to failure performance in the current study. However, low (1%) and moderate (2%) dose of CMR did not show the same effect. This finding supports the occupancy hypothesis [27], that the greater the dosages of caffeine, the more adenosine and/or bitter taste receptors within the mouth may be activated. Speculation can be made regarding which receptor (adenosine vs. taste) was activated in the current study. Considering rinsing with a carbohydrate and caffeine share a common ergogenic mechanism that both of them activate taste receptors that stimulate the brain region (orbitofrontal cortex) related to reward and motor control [40]. The same dose–response relationship was not observed in a previous study showing that low, moderate and high doses of carbohydrate mouth rinse did not increase bench press endurance performance [33]. Thus, it is more likely, in the current study, that a high dose of CMR stimulates adenosine receptors found in the mouth, which increase attention and the release of excitatory neurotransmitters in the brain (dopamine, norepinephrine), exerting their ergogenic action at a central level [14]. However, this is only speculation at this point, because it is not known and measured via electroencephalogram (EEG) which brain regions were activated.

The effectiveness of CMR in a dose-dependent fashion on muscular or aerobic endurance performance has not been examined before; therefore, direct comparisons in this regard cannot be administered. However, a few comparative studies exist that have examined the impact of CMR on exercise performance. Beaven et al. [18] was the first to show that 1.2% of 5 s CMR led to a significant increase in mean power output in the first and second sprints compared with PLA in a test protocol including 5 × 6 s maximal sprints. In another design, 10 s of CMR (2%) increased sprint cycling performance in a low muscle glycogen state [31]. On the contrary, CMR has no effect on the Wingate anaerobic test [41]. Karuk et al. [42] also demonstrated that 300 mg of CMR did not enhance repeated jump performance. Lastly, Clarke et al. [26] concluded that when resistance exercise intensity (60% of 1-RM) elicits near maximal RPE, it creates a “ceiling effect”, which makes any appreciable differences between 300 mg of CMR and placebo extremely hard to distinguish. The current findings partially support the “ceiling effect” assertion because with the same resistance intensity (60% of 1-RM), 750 mg but not 250 or 500 mg of CMR was beneficial, even with similar RPE values as Clarke et al. [26]. In their systematic review, Ferraz da Silva et al. [14] also concluded that only five out of fifteen selected studies found a positive effect with CMR. Differences in data analysis/interpretation, test protocol (resistance vs aerobic type), CMR concentration and rinsing duration [23] may explain these contradictions. Moreover, non-smoking participants performed the test protocol after 10 h night fasting in the early morning in the current study. The activation of the primary taste cortex (insular cortex) in response to a taste stimulation may also be modified by factors such as prandial [43] and smoking status of participants [23]. These two features of our study may potentiate the current HCMR results. CMR appears to be beneficial in enhancing exercise performance to a greater extent under a fast state because most studies showed that no benefits of CMR were performed in a fed state [20,22,26,30,41].

In addition, mouth rinse avoids hepatic metabolism, but Figueiredo et al. [44] did not report an effect of CMR on performance during a 10 km running trial or a vertical jump in individuals’ CC homozygotes or AC heterozygotes. In this regard, inter-individual variation in bitter tasting ability that may modify the CMR responses in the literature can be related to the genotype of participants [17]. Perhaps, in our study, most of the participants had the C allele that may reduce the bitter taste perception [17] and therefore did not respond to the low (250 mg) and moderate (500 mg) doses of CMR. As it is seen, more research is needed to determine the potential factors that may affect the ergogenic effect size of the CMR.

Exposure to caffeine in the mouth enhances brain activity within the dorsolateral and orbitofrontal cortex, regions associated with reward and cognition [40], which could exert a central role in RPE. Significant decrements in RPE values were observed in the HCMR condition. These data are in contrast to those of Melo et al. [21], who showed that CMR increases the fatigue tolerance by the increased RPE and decreased electromyographic (EMG) activity. Some other studies have demonstrated that CMR cannot influence the RPE [17,18,19,20,21,26,41]. RPE responses to CMR are likely to be affected by the dose because these studies that reported no effect on RPE used CMR dosages between 35 and 300 mg. This is consistent with the current results that 250 and 500 mg of CMR did not influence the RPE values. Lastly, no effect of CMR on heart rate values have been consistently reported [14,23], which is in line with the current results that none of the CMR doses had an influence.

The current study is not without limitations. The 48 h resting period was given between test sessions, which cannot be sufficient to ensure full recovery [45]. Moreover, 1-RM strength and three sets of muscular endurance performance were tested in one session. Furthermore, blinding effectiveness was not assessed and the “expectancy” phenomenon may have taken place and impacted the results of the present research. This study was conducted with very low habitual caffeine consumers; thus, current results cannot directly transfer to athletes using high level of daily caffeine. Test sessions were performed in a fasted state, given that muscular endurance training is not commonly performed in a fasted state. Lastly, we did not measure EEG, EMG or blood caffeine or neurotransmitter levels, which would have provided more information to clarify precise mechanisms as to how HCMR enhanced muscular endurance performance and decreased RPE values.

## 5. Conclusions

There were no benefits of CMR on 1-RM strength performance. However, five seconds of mouth rinsing with 750 mg of caffeine improved 60% of 1-RM bench press endurance performance in resistance-trained males. However, the same beneficial effect was not observed with 250 or 500 mg of caffeine mouthwash. Although heart rate did not differ between conditions, RPE values were significantly lower during HCMR. From a practical standpoint, the current study’s results can be used by resistance-trained individuals who perform resistance training in the fasted state to decrease their RPE values while increasing muscular endurance performance. Furthermore, athletes may prefer the practical use of 750 mg of CMR during training in the early morning, instead of caffeine ingestion, which takes an average of 45–60 min to metabolize.

## Figures and Tables

**Table 1:** Heart rate and RPE values.

	PLA		LCMR		MCMR		HCMR	
	M	SD	M	SD	M	SD	M	SD
Heart Rate (Beat/min)								
Pre-Test	63.57	5.13	67.78	5.78	63.85	4.63	63.57	4.76
Post 1-RM	134.85	17.62	133.00	14.30	132.92	14.45	130.50	14.58
1. set	144.64	16.91	150.00	15.52	146.78	14.69	153.85	11.09
2. set	155.92	12.65	156.78	9.72	154.64	11.90	154.50	13.23
3. set	157.57	10.01	159.28	15.61	157.85	12.00	154.35	12.99
Ratings of Perceived Exertion (RPE) (6–20)								
Post 1-RM	14.78	1.52	14.78	1.71	15.42	1.22	15.00	1.24
1. set	18.42	1.39	18.57	1.78	18.21	1.36	17.50 *	1.22
2. set	18.92	1.49	18.78	1.12	18.57	0.85	17.71 *	1.20
3. set	18.92	1.54	19.07	1.59	19.35	0.92	18.00 *	1.56

PLA: placebo; LCMR: low dose of caffeine mouth rinse; MCMR: moderate dose of caffeine mouth rinse; HCMR: high dose of caffeine mouth rinse. *: significantly different from PLA, CLMR and MCMR.

## Data Availability

The data presented in this study are available on request from the corresponding author. The data are not publicly available due to privacy restrictions.
